# Fungous Hæmatodes in the Right Eye, with an Anomalous Tumor at the Base of the Brain

**Published:** 1827-12

**Authors:** Martin Ware


Case II. *Fungous Hæmatodes in the right Eye, with an anomalous Tumor at the Base of the Brain.* By Martin Ware, Esq. With a Drawing, by T. W. Chevalier, Esq.

In April, 1812, Miss M., about forty years of age, consulted my late father on account of a total blindness in the right eye. From a memorandum of the case taken at the time, it appears that she was then suffering from violent pain in the eye, and that the pupil was much dilated.

This lady had for many years been subject to frequent returns of pain in the head, which were not relieved by any mode of treatment that had been adopted.

In June, 1815, she favored me with a visit, when I found that the measures prescribed by my father, three years before, though attended with temporary relief, had been productive of no permanent benefit. The pupil still continued dilated, and the sight was quite destroyed.

For several successive months, I had many opportunities of seeing her, during which time the returns of pain in the head and eye were frequent and violent; but, with the exception of the dilated pupil before mentioned, there was no apparent alteration in the figure or structure of the eye.

In December, 1816, the eye began to enlarge, and soon afterwards the cornea became opaque; the vessels on the globe of the eye being also considerably distended.

In January following, I first met the late Mr. Chevalier in consultation upon the case, and was favored with his assistance on different occasions until the decease of our patient. An operation for the removal of the eye was in the first instance recommended, to which she readily consented: we were, however, induced subsequently to alter our opinion with respect to that advice, upon being informed by her friends that they had perceived an increasing imbecility of her mental faculties, which, in addition to the pain of the head under which she had so long suffered, induced us to fear that the disease was not confined to the eye, and consequently that no permanent benefit would be derived from the proposed operation.

At this time the internal organisation of the eye was entirely destroyed, its place being occupied by a tumor which protruded considerably beyond the boundaries of the socket. The pain in the eye, which before had been only occasional, was now constant, and resisted every means of relief employed. In this state our patient continued until the end of May, when she was suddenly attacked with constant nausea, and frequent vomiting; the tongue being entirely covered with a thick brown fur, and the pulse becoming feeble and variable.

Early in June, she suddenly lost the sight of the left eye, which, as well as the sense of hearing, had till then remained perfect.

She became gradually weaker, and in a few days sunk into a state of coma, in which she continued several days, and expired on the 11th of August.

*Dissection.*—On raising the anterior right lobe of the cerebrum, a soft, irregularly shaped, dark-red tumor was seen to proceed from the roof of the orbit, near the crista galli, and to extend backwards over the sella turcica, having in its progress totally disorganised the optic nerves at their decussation. On tracing it, it was found to extend into the substance of the thalami, giving a rotten appearance to the cerebral substance in its vicinity. Inferiorly, it terminated almost immediately before the mammillary processes. At first we had no doubt that this tumor proceeded from the diseased eye, and that we should find the orbit carious; but subsequent examination showed this presumption to be erroneous. The orbit was perfectly sound, and the tumor was attached to, and appeared to have proceeded from, the dura mater only, extending backward as it grew.

The disease within the orbit was confined to the globe of the eye itself, and contained within its coats. The optic nerve, at its entrance into the eye, was sound, but shrunk; and the muscles of the eye and upper eyelid, though pressed close against the periosteum by the enlargement of the eyeball, were separable along their whole course by very easy dissection, after the bone was broken away. A vertical section of the globe itself, from near the outer canthus of the orbit backward, showed the humors to have been entirely removed, and their place supplied by a fungous mass, consisting of many small vascular portions blended together, some of a dark colour, and not unlike the parenchyma of the liver in substance; others were of a whitish hue, resembling brain.

*Explanation of the Plate.*

Figure 1, represents the diseased mass contained within the right orbit. On its upper margin is seen a small portion of the tumor within the cranium; beneath this, the walls of the orbit surrounding the fungous mass which occupied the globe of the eye. The protrusion of the eyeball is delineated on the one side, and on the other the optic nerve is represented considerably diminished in size, but not otherwise changed.

Figure 2, represents a portion of the tumor contained within the cranium. At the upper part of this drawing are seen the corpora quadrigemina, under which is a portion of bougie in the iter ad infundibulum; and another a little lower in the aqueduct of Sylvius. Beneath these objects, the substance of the brain appears considerably changed, especially upon its remaining diseased surface, which is attached to the morbid and unnatural growth.

The tumor is seen covered at the upper part, in irregular patches, with an extremely delicate membrane, more nearly resembling a spider's web than the arachnoid itself; at the lower part, the internal structure of the tumor is exposed, consisting of turgid vessels and loose shreds of membrane, with extravasated blood in their interstices. The whole mass, when complete, was as large as a goose's egg.

